# A Comprehensive Analysis of the Validity and Reliability of the Perception Neuron Studio for Upper-Body Motion Capture

**DOI:** 10.3390/s22186954

**Published:** 2022-09-14

**Authors:** Yiwei Wu, Kuan Tao, Qi Chen, Yinsheng Tian, Lixin Sun

**Affiliations:** 1 AI Sports Engineering Lab, School of Sports Engineering, Beijing Sport University, Beijing 100084, China; 2 Sports Engineering Research Center, China Institute of Sport Science, Beijing 100061, China

**Keywords:** inertial motion capture system, upper-body kinematics, biomechanics, validity, reliability

## Abstract

The Perception Neuron Studio (PNS) is a cost-effective and widely used inertial motion capture system. However, a comprehensive analysis of its upper-body motion capture accuracy is still lacking, before it is being applied to biomechanical research. Therefore, this study first evaluated the validity and reliability of this system in upper-body capturing and then quantified the system’s accuracy for different task complexities and movement speeds. Seven participants performed simple (eight single-DOF upper-body movements) and complex tasks (lifting a 2.5 kg box over the shoulder) at fast and slow speeds with the PNS and OptiTrack (gold-standard optical system) collecting kinematics data simultaneously. Statistical metrics such as CMC, RMSE, Pearson’s r, R^2^, and Bland–Altman analysis were utilized to assess the similarity between the two systems. Test–retest reliability included intra- and intersession relations, which were assessed by the intraclass correlation coefficient (ICC) as well as CMC. All upper-body kinematics were highly consistent between the two systems, with CMC values 0.73–0.99, RMSE 1.9–12.5°, Pearson’s r 0.84–0.99, R^2^ 0.75–0.99, and Bland–Altman analysis demonstrating a bias of 0.2–27.8° as well as all the points within 95% limits of agreement (LOA). The relative reliability of intra- and intersessions was good to excellent (i.e., ICC and CMC were 0.77–0.99 and 0.75–0.98, respectively). The paired *t*-test revealed that faster speeds resulted in greater bias, while more complex tasks led to lower consistencies. Our results showed that the PNS could provide accurate enough upper-body kinematics for further biomechanical performance analysis.

## 1. Introduction

Optical motion capture systems such as Vicon (Oxford, UK) and OptiTrack (Natural Point, Corvallis, OR, USA) are commonly employed as the gold standard to analyze biomechanical performance in the field of sports biomechanics [1,2,3]. Even though these systems have been extensively used, they are prohibitively expensive, inconvenient, and require a laboratory environment [4]. Meanwhile, with the technological advancements in IMU manufacturing, IMU-based motion capture systems offer a low-cost, portable alternative for analyzing biomechanical performance in clinical and sports settings [5]. Current commercial IMU-based motion capture systems, such as Xsens (Xsens Technologies, Enschede, The Netherlands), BioSyn (Surrey, BC, Canada), ImeasureU (Auckland, New Zealand), and APDM (Opal brand, Portland, OR, USA), have adopted a small form-factor IMU, which could be easily secured to body parts with elastic nylon straps. However, these commercial devices exhibit various price points and degrees of validity and reliability. Therefore, novel IMU-based motion capture systems must be validated before being applied to movement technique analysis and clinical rehabilitation [6].

The accuracy of these commercial inertial motion capture systems for measuring upper-body, lower-body, and full-body kinematics has been validated to varying degrees [7,8,9,10]. As one of the most popular IMU-based commercial motion capture systems, the Perception Neuron Studio (PNS, Noitom, Beijing, China) offers a cost-effective (i.e., relative price advantage vs. OptiTrack) and user-friendly (i.e., simple setup and post-processing procedures vs. OptiTrack) option for application in the biomechanical field, and several studies have been performed on its accuracy of kinematic measurements. Sers et al. [11] analyzed the validity of the Perception Neuron system, also from Noitom, in upper-body kinematic analysis by comparing it with the Vicon. The results indicated that the Perception Neuron system was valid enough for assessing most upper-body kinematics, with all multiple correlation coefficients (CMC) equaling 0.99. Choo et al. [12] also analyzed the validity of the Perception Neuron system in full-body motion capture by comparing it with the Vicon. The results showed that most joint angles had an average RMSE of less than 4°, and the Pearson correlation coefficient was higher than 0.85, indicating that the Perception Neuron system is sensitive enough to measure changes in joint angles. In addition, Shuai et al. [13] compared the mean difference in lower-body joint angles between the PNS and OptiTrack systems, with the results showing that RMSE ranges from 3.57° to 13.14°, while CMC values are within 0.47 to 0.99, suggesting that the PNS could evaluate lower extremity kinematics.

Despite the fact that several studies have been conducted to verify the accuracy of these commercial motion capture systems in quantifying kinematics, some problems still exist. First, current studies targeting the upper body are absent from addressing all joints and planes of motion, as are comprehensive validation studies of the three-dimensional (3D) joint kinematics of upper-body movements. Since the measurement of upper-body kinematics is more complex than that of the lower body [14], more challenges remain. Second, as with other commercial motion capture systems [9,15,16,17], almost no studies focus on the reliability of the PNS. Meanwhile, the reproducibility of joint angles for the same movement of subjects has been a clinical issue. Furthermore, the accuracy of IMU-based motion capture systems has been reported to be affected by the complexity of the task and the movement speed [14,18], as these systems are also affected by ferromagnetic interference [19] and drift errors due to signal integration [20,21], requiring fusion algorithms to provide accurate data. These issues have also not been fully resolved in existing studies.

The main contributions of this work are twofold. First, the validity and intra/intersession reliability of upper-body kinematics of the PNS are analyzed by comparing to the gold-standard optical motion capture system. Second, this study provides an in-depth analysis of the effect of task complexities and movement speeds on PNS accuracy, which stimulates further investigations into the accuracy of inertial motion capture systems.

## 2. Materials and Methods

### 2.1. Participants

Seven healthy students (seven males, age: 23.28 ± 1.25 years, height: 176.14 ± 8.01 cm, body mass: 76 ± 7.46 kg) from Beijing Sport University participated in this study, after signing an informed consent form. The study was permitted by the Ethics Committee of Beijing Sport University (2022108H) and adhered to the Declaration of Helsinki. The exclusion criteria were that participants had no musculoskeletal or chronic neurological disorders and were able to autonomously perform all tasks.

### 2.2. Instrumentation

**Figure 1:**
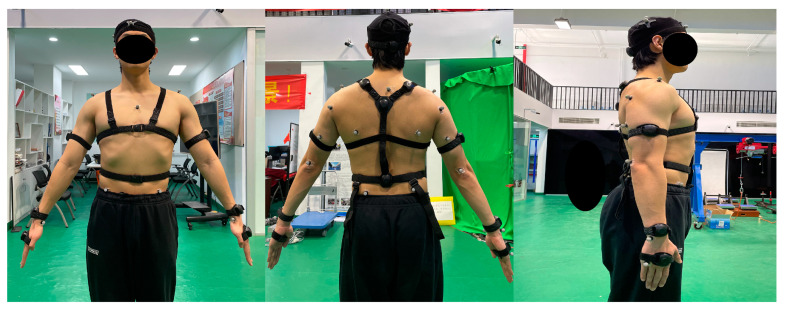
Placements of 11 IMUs and 25 reflective markers on a participant.

Upper-body kinematics were collected simultaneously at 100 Hz by the PNS and the OptiTrack with 10 high-speed cameras. Eleven IMUs (15.5 g; 43 × 33 × 20 mm) and twenty-five reflective markers (diameter of 14 mm) were placed on the participants’ body parts as shown in Figure 1. In accordance with the recommendations of the manufacturer [22], the PNS IMUs were placed on the back of the head, shoulders (upper portion of the scapula), upper spine, lower spine (just above the hips), upper arms (above the lateral elbow), and forearms (just above the lateral side of the wrist). A three-axis accelerometer (32 g), a three-axis gyroscope (2000 DPS), and a three-axis magnetometer were included in each IMU. Reflective markers were affixed to anatomical landmarks with medical pressure-sensitive adhesive tape, and the position of these reflective markers was independent of the positions of the IMUs. Based on the OptiTrack baseline upper-body marker sets [23], the reflective markers were made to be used in OpenSim (Stanford University, Stanford, CA, USA, version 4.3) [24].

### 2.3. Experimental Protocol

The calibration was performed in accordance with the user guides of the manufacturer for PNS [22] and OptiTrack [23], respectively. Static calibration data were collected before the start of each experimental protocol to scale the OpenSim full-body model, while anthropometric data were collected to adjust the PNS biomechanical model. Before each test session, the site must be cleared of metal objects to avoid any impact on the PNS accuracy. Participants were required to perform both simple and complex tasks at fast and slow speeds to assess the accuracy of upper-body kinematics provided by the PNS, with the simple task defined as movements occurring in only one motion plane, while the complex task occurred in multiple planes. The simple task included 8 single-DOF (degree of freedom) movements of the trunk (3), shoulder (3), and elbow (2), i.e., thorax flexion/extension, thorax lateral flexion, thorax rotation, shoulder flexion/extension, shoulder adduction/abduction, shoulder internal/external rotation, elbow flexion/extension, and forearm pronation/supination. The complex task was to lift a 2.5 kg box over the shoulder [25]. To verify intrasession reliability, each participant was required to perform each task three times within one test session. Similarly, two sessions of testing were required to verify the intersession reliability, with the sensors being reworn, reflective markers retaped, and the PNS and OptiTrack recalibrated before each session. Additionally, a full test would start from the participant’s anatomical position, and all trials would proceed in order.

### 2.4. Data Preprocessing

Marker track reconstruction and automated marking were initially performed with Motive (version 2.2.0, OptiTrack Inc., Corvallis, OR, USA) for the marker data collected by the OptiTrack. Each trial was then visually inspected, and the unmarked trajectories were manually labeled and exported as a .trc file. The data were then filtered by a 4th-order Butterworth low pass filter (6 Hz) to remove any high-frequency noise before being imported into a 42-DOF skeletal model in OpenSim [26]. The model was scaled to the participant using static reflective markers data, and joint angles were estimated based on inverse kinematics [27] that minimized the least squared distance between each pair of experimental and model markers [28], as shown in Equation (1).

(1) $$\underset{q}{min}\left[\sum_{i\in maekers}w_{i}{\left(x_{i}^{exp}-x_{i}^{model}\right)}^{2}+\sum_{j\in coords}w_{i}{\left(q_{j}^{exp}-q_{j}\right)}^{2}\right],$$

where q is the vector of generalized coordinates being solved for, $x_{i}^{exp}$ is the experimental position of marker i, $x_{i}^{model}$ is the corresponding model marker (which depends on the coordinate values), $q_{j}^{exp}$ is the experimental value for coordinate j, and $q_{j}$ is the coordinate value of the model of all the designated j points [29]. The raw data collected by PNS were processed and exported to skeletal quaternions using Axis Studio (Noitom, Beijing, China, version 2.5). The joint angle quaternions were computed based on Equation (2), and the upper-body joint angles were calculated according to the YXZ rotation order [30].

(2) $$q_{joint}={\left(q_{r}{}^{b1}\right)}^{-1}\;⊗\;q_{r}{}^{b2},$$

where $q_{joint}$ is the target joint quaternion, $q_{r}{}^{b1}$ is the distal segment limb quaternion, $q_{r}{}^{b2}$ is the proximal segment limb quaternion, and ⊗ denotes the quaternion multiplication. To be noted, the Euler angles adopted in OpenSim are in XYZ rotation order [26], which is not consistent with the PNS. As a result, the upper-body kinematics from the two systems were processed for consistency before further investigation. Furthermore, the angular curves from the two systems were then synced using a peak detection algorithm, and the joint angle time series of all trials were also normalized to 100 frames to allow for parallel comparisons.

### 2.5. Statistical Analysis

To evaluate the validity of the upper-body kinematics provided by the PNS through comparison with the OptiTrack system, several statistical metrics, i.e., coefficient of multiple correlation (CMC) [31,32], root mean square errors (RMSE), and Pearson’s r were calculated. The CMC and Pearson’s r and coefficient of determination (R²) were calculated to evaluate the curve similarity and the RMSE between curves collected by the two systems as an overall measure of curve consistency. Additionally, Bland–Altman analysis [33] was used to characterize the agreement between the two systems through the calculation of the mean difference (bias) and 95% limits of agreements (LOA).

Test–retest reliability refers to both intrasession and intersession relations. Therefore, the intraclass correlation coefficient (ICC) [34] with a two-way mixed model was used to evaluate the intrasession reliability, whereas the ICC with a two-way random model as well as CMC was calculated to assess the intersession relative reliability. Furthermore, the typical error of measurement (TEM), the smallest worthwhile change (SWC), and the minimal detectable change (MDC) [35] were used to assess the intersession absolute reliability. The ability of the test to detect a change was rated as “good”, “OK”, or “marginal” when the TEM was below, similar to, or higher than the SWC, respectively. SWC was considered as typical small effect (SWC_0.2_), moderate effect (SWC_0.6_), and large effect (SWC_1.2_). The MDC_95%_ of upper-body range of motion, which represents 95% CI of the difference in score between paired observations, was determined as ${\mathrm{MDC}}_{95\%}=\mathrm{TEM}\times 1.96\times \sqrt{2}$ [36].

CMC, Pearson’s r, R², and ICC were considered as excellent (>0.90), good (0.75–0.90), moderate (0.5–0.75), and poor (<0.50) [37]. RMSE of 5° to 10° was considered good, while RMSE < 5° was excellent [38]. The effects of task complexities and movement speeds on the accuracy of the PNS were tested for significance using paired *t*-tests, and a Shapiro–Wilk test was carried out to test the normality of the data distributions. The statistical significance level was set at 5%. The magnitude of the significant changes was analyzed using Cohen’s effect sizes: small effect (d ≥ 0.2), medium effect (d ≥ 0.5), and large effect (d ≥ 0.8). The statistics were calculated using Python (version 3.9) and SPSS (IBM Corporation, Armonk, NY, USA, version 26.0).

## 3. Results

The kinematics of 756 upper-body functional movements (seven participants, 27 movements, two speeds, two test sessions) were analyzed in this study. Simple tasks, flexion and extension, adduction and abduction, internal and external rotation, and forearm pronation and supination, were considered separately for the consistency analysis. In all tasks investigated, the data from both sides were averaged, considering that the movements of the left and right sides were balanced [39].

### 3.1. PNS’ Concurrent Validity in Upper-Body Assessment

**Figure 2:**
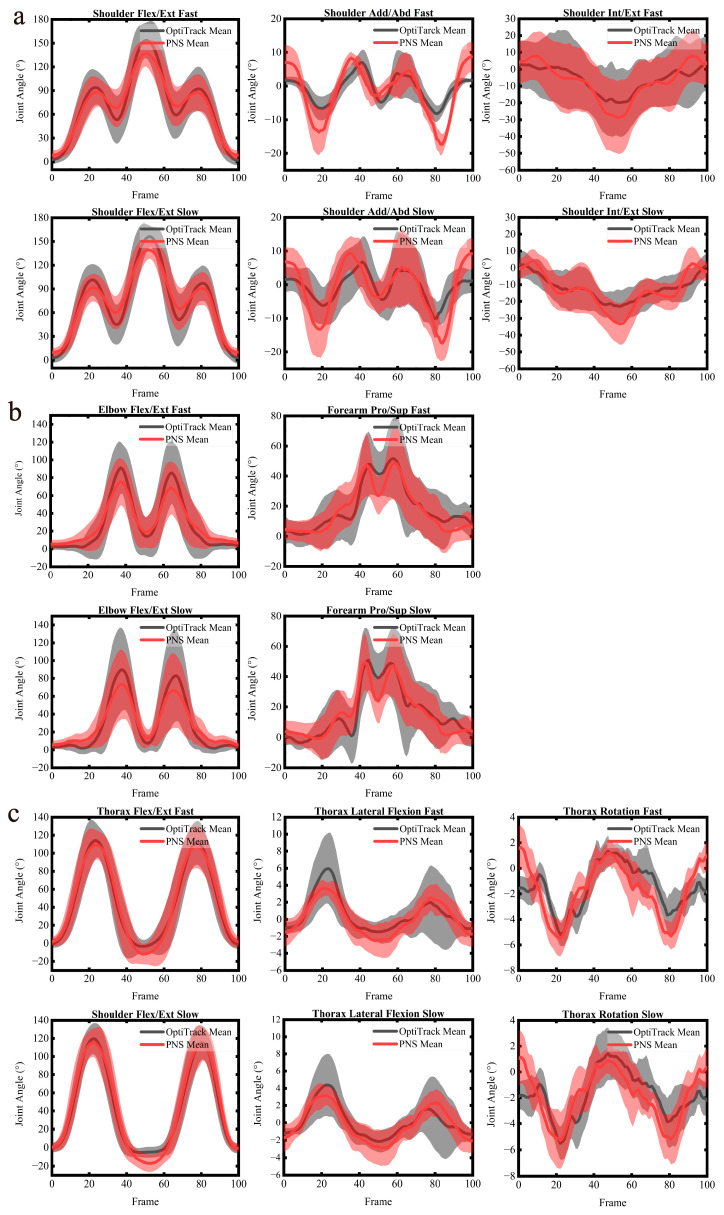
Comparison of the PNS and OptiTrack’s mean angular curves during the complex task. (**a**–**c**) The mean angular curves of the shoulder (**a**), elbow (**b**), and trunk (**c**). The thick solid line represents the mean angular curve for the PNS (red) and OptitTrack (black), and the shadow area represents standard deviations from the mean angular curve. Note that Add/Abd, Flex/Ext, Int/Ext, and Pro/Sup stand for adduction/abduction, flexion/extension, internal/external rotation, and pronation/supination, respectively.

**Figure 3:**
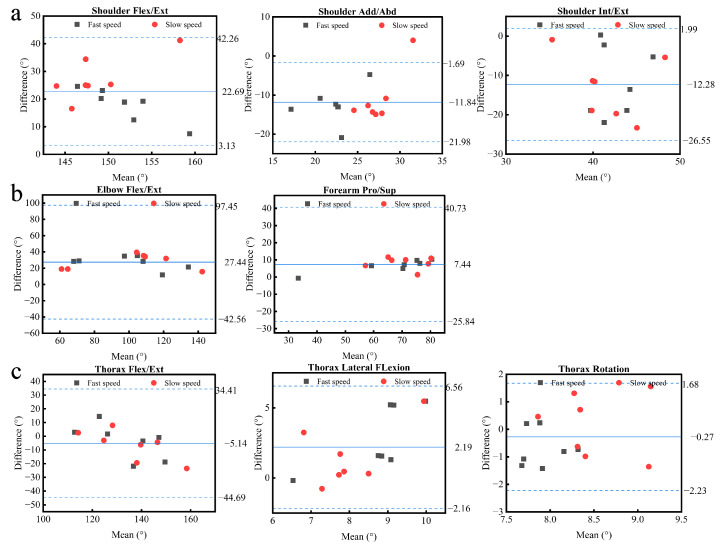
Bland–Altman plots for each absolute angular of the shoulder (**a**), elbow (**b**), and trunk (**c**) in the complex task. The solid blue horizontal line stands for the mean bias, while the dashed blue horizontal lines indicate the upper and lower 95% confidence intervals. Note that Add/Abd, Flex/Ext, Int/Ext, and Pro/Sup stand for adduction/abduction, flexion/extension, internal/external rotation, and pronation/supination, respectively.

**Figure A1:**
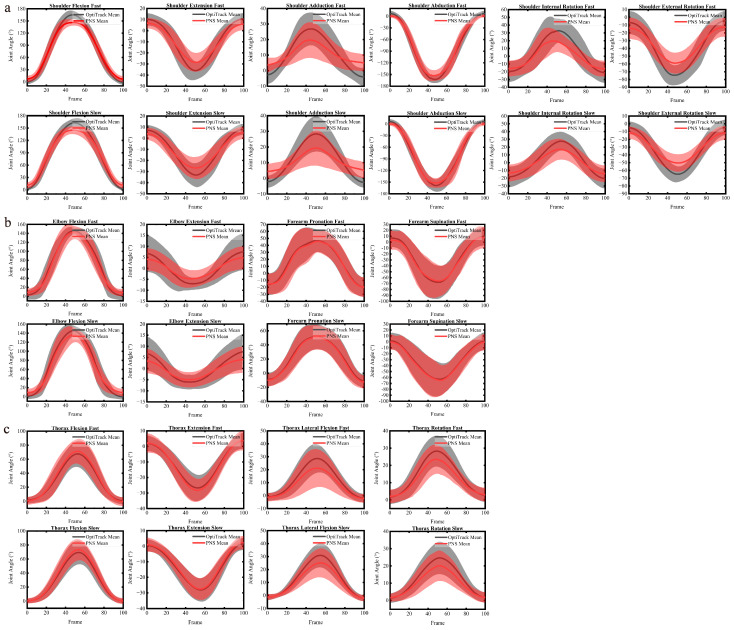
Comparison of the PNS and OptiTrack’s mean angular curves during simple tasks. (**a**–**c**) The mean angular curves of the shoulder (**a**), elbow (**b**), and trunk (**c**). The thick solid lines represents the mean angular curve for the PNS (red) and OptitTrack (black), and the shadow area represents standard deviations from the mean angular curve.

**Figure A2:**
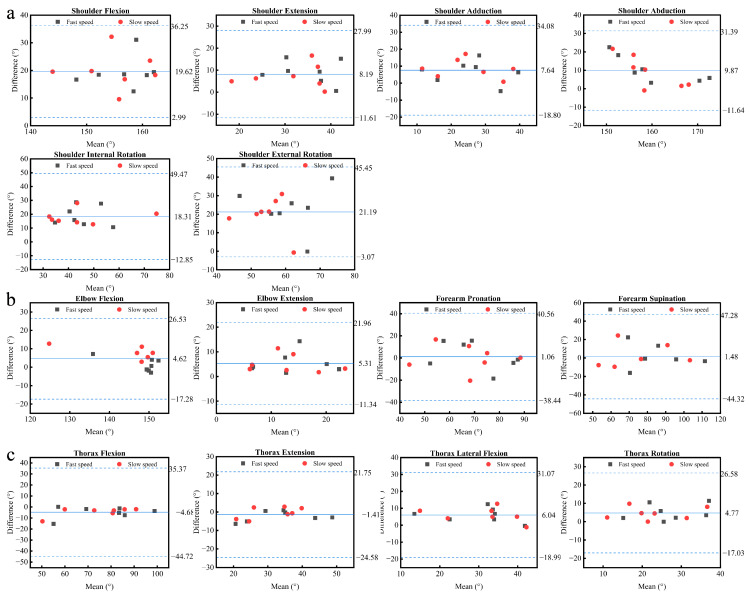
Bland–Altman plots for each absolute angular of the shoulder (**a**), elbow (**b**), and trunk (**c**) in simple tasks. The solid blue horizontal line stands for the mean bias, while the dashed blue horizontal lines indicate the upper and lower 95% confidence intervals.

Figure 2 and Figure 3 show the comparison of the upper-body motion angular curves between the PNS and OptiTrack for the complex task and the Bland–Altman plot of the upper-body range of motion, respectively, while simple tasks are shown in the Appendix A Figure A1 and Figure A2. The metrics for validity (i.e., CMC, RMSE, Pearson’s r, R^2^, and LOA) of the PNS are presented in Table 1 and Table 2.

Most upper-body angular curve consistency was good to excellent during all investigated tasks between the two systems, with CMC values ranging from 0.78 to 0.99, except for shoulder adduction, which had an average CMC of 0.73. In comparison with the shoulder and elbow, the trunk angular curves demonstrated the highest degree of consistency, with CMC values ranging from 0.90 to 0.96. Furthermore, the correlation for most upper-body angular curves between the two systems was excellent, with Pearson’s r values ranging from 0.90 to 0.99. When comparing the trunk to the shoulder and elbow, the trunk had the strongest correlation between the two systems, with Pearson’s r and R² values between 0.84 and 0.99. The weakest correlation was found in shoulder adduction, with an average Pearson’s r of 0.85 and R^2^ of 0.76.

For all upper-body movements across all investigated tasks, the RMSEs between the angular curves from the two systems ranged from 1.9° to 12.5°. The relatively large errors were found in shoulder abduction and elbow flexion/extension, with an average RMSE value of 11.3° and 12.4°, respectively. Except for thorax flexion/extension, which had an average RMSE of 10.2°, the trunk showed the smallest disparity (i.e., RMSE < 5.0°) between the two systems. The Bland–Altman analysis indicated that the angular curve range of motion (ROM) differences (bias) between the two systems ranged from 0.2° to 27.8°, with limits ± 1.3° to ± 25.8° across all investigated tasks. The elbow flexion/extension demonstrated the greatest mean bias of 27.5° for the complex task. When compared to the other joints, the thorax rotation had the smallest mean difference, with an average bias of 0.5°. Furthermore, Bland–Altman plots showed that all the points were within the 95% LOA.

### 3.2. PNS’ Intra- and Intersession Reliability

The intra- and intersession relative reliability metrics of the PNS are presented in Table 1 and Table 2 (i.e., the values of ICC and CMC), while the absolute reliability metrics are presented in Appendix A, Table A1. Across all investigated tasks, all angular curves demonstrated good to excellent intrasession reliability as well as intersession relative reliability, with ICC values ranging from 0.77 to 0.99 and CMC values between 0.75 and 0.98. Among the upper-body parts investigated, the trunk angular curves exhibited the highest reliability, with ICC values ranging from 0.86 to 0.98 and CMC values from 0.86 to 0.95, whereas the elbow showed the lowest reliability, with ICC values ranging from 0.77 to 0.98 and CMC values from 0.75 to 0.95. Elbow extension was the least reliable, with a mean intrasession ICC value of 0.78 and intersession ICC and CMC values of 0.79 and 0.75, respectively, while shoulder flexion was the most reliable, with a mean intrasession ICC value of 0.98 and intersession ICC and CMC values of 0.99 and 0.98, respectively. Furthermore, all TEMs were below or similar to SWC_0.2_, which showed higher intersession absolute reliability and a good ability of the PNS to detect smaller performance changes.

### 3.3. Task Complexity and Movement Speed Analysis

**Figure 4:**
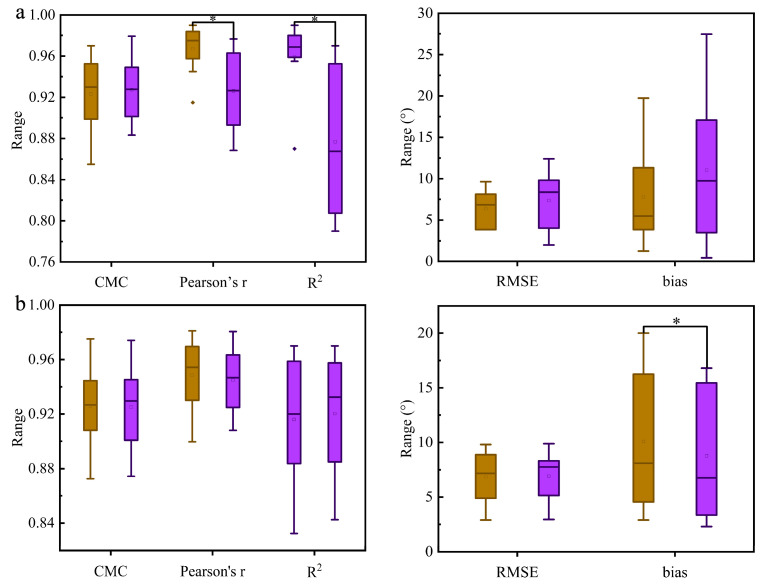
Box-and-whisker plots of the validity metrics (i.e., CMC, Pearson‘s r, RMSE, and bias). (**a**) simple (brown) and complex (purple) tasks. (**b**) Fast (brown) and slow (purple) speeds. Note that ∗ represents p<0.05.

The Shapiro–Wilk test indicated that the distributions of the differences in validity metrics between the simple and complex tasks, as well as between the fast and slow ones, were normal (*p* < 0.05). In terms of the task complexity, except for Pearson’s r (*p* = 0.029, Cohen’s d = 0.969) and R² (*p* = 0.015, Cohen’s d = 1.14), the outcomes of the paired *t*-test with a significance value of 5% showed no significant differences between the simple and complex tasks (Table 3 and Figure 4) in terms of validity metrics. The average for the simple task’s Pearson’s r was 0.97, whereas the overall mean for the complex task was 0.93. Furthermore, there were no significant differences in the validity metrics between the fast and slow speeds in terms of movement speeds (Table 3 and Figure 4), except for bias (*p* = 0.046, Cohen’s d = 0.855). The slow speed had a mean bias of 8.2°, while the fast speed had a mean of 10.1°.

## 4. Discussion

The primary purpose of this investigation was first to evaluate the validity and intra/intersession reliability of upper-body kinematics of the PNS in comparison with the OptiTrack (gold standard) and then to quantify the system’s accuracy at different task complexities and movement speeds. Our results demonstrated that the PNS had a high-level accuracy in measuring upper-body kinematics with validation by the OptiTrack. We also discovered that the intra/intersession reliability of the PNS in the majority of the upper-body angular curves was high. In addition, we found faster speeds resulted in larger bias and more complex tasks led to lower consistency, while the differences were small.

### 4.1. PNS’ Concurrent Validity in Upper-Body Assessment

The PNS and OptiTrack angular trajectories showed similar curve patterns in all upper-body movements across all investigated tasks. This was especially true in the sagittal plane, where CMC values ranged from 0.78 to 0.99, and Pearson’s r was between 0.95 and 0.99, which was similar to the results of lower extremity research of the PNS [13], indicating that the PNS has better accuracy in motion capture in the sagittal plane. Furthermore, the complex task of this study was to lift a 2.5 kg box over the shoulder, with most of the movement occurring in the sagittal plane. This indicated that the PNS performed better in the main motion plane than in other planes, which was consistent with what other prior studies [40,41] of commercial motion capture systems found.

Nevertheless, the RMSEs that were found between the kinematic patterns of shoulder adduction measured by the two systems were not consistent with the angular curve agreements. The angular curve’s CMC indicated moderate consistency, with a mean CMC value of 0.73; in contrast, the RMSE was below 10°, which suggested that the consistency was good. This inconsistency could be explained by the fact that the displacement of the IMU placed on the upper arm varied less than the reflective marker throughout the shoulder adduction movement, which resulted in a lower consistency when compared to the gold standard. Furthermore, the largest errors were observed in the elbow flexion, with an average RMSE of 12.4°, which would benefit from a functional motion axis setup. The correct placement of the IMU was made more difficult by the fact that forearm pronation/supination movements could rotate the flexion/extension axis of the IMU, causing it to be unaligned with the anatomical flexion/extension axis of the elbow [42]. In general, the mean errors between the two systems’ evaluations of angular curves were greater for tasks or motion planes involving a larger range of motion. This was in line with the findings reported by Mavor et al. [43].

Previous validation studies [11,12] of the Perception Neuron system had reported better angular waveform similarity in the trunk than in the shoulder and elbow, compared to the gold standard. In the present research, we also reached the identical conclusion, that the angular curve of the trunk measured by the PNS was more consistent with the reference system than the shoulder and elbow. This finding was also consistent with, and in many cases outperformed, prior research comparing IMU-based motion capture systems to optical systems [12,44,45,46,47,48]. In comparison to a study by Shuai et al. [13], the accuracy presented by the PNS in this investigation in measuring the upper-body kinematics was slightly lower than that of the lower body, which was also consistent with the results of some studies [9,14]. This could be because the movements of the upper body usually revolve around two or three axes, whereas the movements of the lower body mostly take place in closed chains and one plane of motion.

According to the Bland–Altman analysis, the average systematic bias for most joints between the two systems was below 10°, except for the shoulder flexion, shoulder external rotation, shoulder internal rotation for simple tasks, shoulder flexion/extension, shoulder adduction/abduction, shoulder internal/external rotation, and elbow flexion/extension for the complex task. In general, the PNS underestimated joint angles for most wide ranges of motion and overestimated joint angles for most minor ranges of motion when compared to the gold standard, which had been similarly reported in other validation studies [42,49,50]. Additionally, Bland–Altman plots showed that all points were within 95% of the LOA, indicating that the PNS and OptiTrack were generally consistent.

As discussed above, upper-body kinematics assessed by the PNS exhibited good to excellent consistency with OptiTrack, while it was obvious that the PNS struggled to provide sufficiently accurate kinematic data for shoulder adduction movement. Reflective marker placement on anatomical markers rather than attached to the IMU, which was the preferred method in some studies [51,52] to minimize errors, might account for some of the observed discrepancies between the two systems in this investigation. Finally, participants were requested to stand in the calibrated posture of A and T before each session. Mismatches between the actual marker position and the model position can also result in errors in joint angle estimates [53].

### 4.2. PNS’ Intra- and Intersession Reliability

Like optical systems, IMUs are susceptible to the effects of artifacts induced by the displacement of the sensor and underlying tissue relative to the bone [54]. This artifact is often referred to as a “soft tissue artifact” (STA) [52], in which measurement errors are caused by soft tissue slippage and vibration that occurs intrasession (test within one calibration) at random. However, we found high intrasession reproducibility of the PNS-measured upper-body angular curves with ICC values of 0.78–0.98 across all tasks investigated, indicating that soft tissue artifacts have a negligible effect on the intrasession test reliability of the PNS.

When comparing the data from the intersession test, we discovered that the CMC and ICC for all upper-body angular curves assessed by the PNS varied from good to excellent, and all TEMs were below or similar to SWC_0.2_. In comparison to the other planes, the sagittal plane demonstrated the highest relative and absolute reliability, consistent with previous findings [55,56], which indicated that flexion/extension movements were more reliable than abduction and rotation. The worst relative reliability was found in elbow flexion (i.e., ICC = 0.79 and CMC = 0.75) and shoulder adduction (i.e., ICC = 0.82 and CMC = 0.76), and such a phenomenon might be attributed to the sensor wearing position and the instability of the motion itself. Furthermore, the number of studies on the reliability of the upper body is small compared to the lower body [14]. As a result, our findings complemented the reliability results of other studies, with CMC and ICC ranging from good to excellent in all planes across all tasks, and the PNS had the ability to detect smaller performance changes.

Overall, the PNS demonstrated clinically acceptable repeatability throughout, with the exceptions of the shoulder adduction and elbow extension values, which need to be interpreted with caution due to their potentially misleading nature.

### 4.3. Task Complexity and Movement Speed Analysis

We found that faster movement speeds resulted in greater bias, and more complex tasks led to lower consistencies. However, this difference was small, as most validity metrics were not significantly different (*p* > 0.05). Previous studies [14,18] also reported that the complexity of the task and the speed of movement can have an impact on the accuracy of the IMU. Similarly, the research observed by Sers et al. [11] on the validity of the Perception Neuron system also found that movement speeds made a significant difference in bias compared to the optical system. Furthermore, Nüesch et al. [57] found that running had a larger RMSE than treadmill walking, with mean errors below 8° and 5°, respectively. A review by Cuesta-Vargas et al. [38] also reported that more complex tasks would decrease validity, although most of the referenced research only tested movements that occurred in a single plane, such as isolated flexion and extension. Our results were similar to those of other investigations and extended the previous findings. In general, the PNS exhibited a high level of consistency with OptiTrack across all tasks when measuring the upper-body kinematics. However, the accuracy of the PNS was slightly affected by increased task complexities and movement speeds, so care should be taken to interpret the relevant measurements in practical applications.

### 4.4. Limitation and Future Work

There are three main limitations to this study. First, the sample size for this study was limited to only seven Beijing Sports University students, and the PNS has not been used in patients with upper-body dysfunction in a clinical setting. Upper-body movements in healthy individuals tend to have low variability and will likely lead to higher validity and reliability. Therefore, a validation study with a larger and more diverse sample size will be conducted in the future.

Second, IMU-based inertial motion capture systems have some obvious limitations, including ferromagnetic interference sensitivity and signal integration drift problems, which require fusion algorithms to provide reliable kinematic data [20,58]. Although the site of ferromagnetic interference objects was cleared before the experiment, the drift error due to the gyroscope signal integration over time was not considered in this study. In the future, longer experiments will be done to find out if the manufacturer’s proprietary algorithms or sensors have any limitations that would affect the accuracy of the kinematic data captured by the PNS when used over a long period of time.

Finally, we investigated the impact of movement speeds on the accuracy of kinematic data captured by the PNS. However, there are still some shortcomings. In our study, we found that fast trials resulted in greater bias than slow trials, but the speed of movement did not have a significant effect on the consistency of the angular waveforms between the two systems. This may be due to the fact that the speed of movements in this investigation was self-selected by the participants. Therefore, further studies could adopt the multi-model approaches [59,60,61,62] to explore the effect of movement speeds on the accuracy of PNS measurement in more details.

## 5. Conclusions

With the investigation of the validity and reliability of the PNS in terms of measuring upper-body kinematics during simple and complex tasks, the PNS was proved to be capable of accurately quantifying upper-body kinematics in different tasks regardless of task complexities and movement speeds. In general, the PNS has great potential as a low-cost, portable, and user-friendly option for assessing upper-body angular curves during functional tasks. Thus, the PNS could provide accurate enough upper-body kinematics for further biomechanical performance analysis. Furthermore, it could also help doctors or physiotherapists diagnose and treat patients more quickly in the clinical field, while, in the sports field, it could help coaches evaluate athletes more effectively to improve their training and also promote sports health development for non-athletes.

## Figures and Tables

**Table 1:** The statistical metrics of validity and reliability of the PNS in simple tasks.

Variable			Validity						Reliability			
			Degree of Validity	CMC	RMSE (°)	Pearson’s r	R^2^	LOA (°)	Degree of Reliability	ICC (Intrasession)	ICC (Intersession)	CMC (Intersession)
Shoulder	flexion	Fast	excellent	0.99	8.8	0.99	0.99	19.3 ± 11.2	excellent	0.98	0.99	0.98
		Slow	excellent	0.98	9.2	0.99	0.99	20.0 ± 13.4	excellent	0.98	0.99	0.98
	extension	Fast	excellent	0.95	4.1	0.98	0.97	9.1 ± 10.6	excellent	0.92	0.95	0.89
		Slow	excellent	0.96	3.4	0.98	0.97	7.3 ± 10.5	excellent	0.94	0.96	0.88
	adduction	Fast	moderate	0.73	8.4	0.84	0.75	8.5 ± 10.9	good	0.81	0.84	0.75
		Slow	moderate	0.73	7.6	0.85	0.77	6.8 ± 13.1	good	0.82	0.79	0.77
	abduction	Fast	good	0.98	11.1	0.98	0.98	10.5 ± 14.3	excellent	0.98	0.98	0.98
		Slow	good	0.98	11.4	0.99	0.98	9.3 ± 17.1	excellent	0.98	0.98	0.98
	internal rotation	Fast	excellent	0.92	9.3	0.98	0.96	18.8 ± 14.4	excellent	0.95	0.94	0.84
		Slow	excellent	0.90	7.4	0.98	0.97	17.8 ± 10.2	excellent	0.90	0.92	0.85
	external rotation	Fast	excellent	0.91	8.9	0.98	0.98	22.7 ± 23.6	excellent	0.96	0.96	0.91
		Slow	excellent	0.91	8.1	0.99	0.97	19.7 ± 19.7	excellent	0.93	0.97	0.93
Elbow	flexion	Fast	excellent	0.98	8.8	0.98	0.98	1.3 ± 7.2	excellent	0.97	0.97	0.94
		Slow	excellent	0.98	8.7	0.99	0.99	7.9 ± 6.5	excellent	0.98	0.97	0.95
	extension	Fast	good	0.78	6.0	0.96	0.93	5.5 ± 8.5	good	0.78	0.78	0.75
		Slow	good	0.81	5.8	0.95	0.92	5.1 ± 7.2	good	0.78	0.79	0.75
	forearm pronation	Fast	excellent	0.96	7.6	0.99	0.98	1.9 ± 25.2	good	0.91	0.92	0.88
		Slow	excellent	0.96	7.2	0.99	0.99	0.2 ± 23.8	excellent	0.91	0.94	0.87
	forearm supination	Fast	excellent	0.95	8.6	0.97	0.95	1.9 ± 24.4	good	0.83	0.81	0.83
		Slow	excellent	0.95	7.8	0.97	0.95	1.1 ± 25.8	good	0.77	0.84	0.81
Thorax	flexion	Fast	excellent	0.96	4.2	0.97	0.96	−4.9 ± 10.2	good	0.88	0.98	0.95
		Slow	excellent	0.96	4.3	0.97	0.96	−4.4 ± 7.7	excellent	0.96	0.97	0.95
	extension	Fast	excellent	0.94	3.3	0.97	0.96	−2.3 ± 5.5	excellent	0.93	0.96	0.93
		Slow	excellent	0.94	3.6	0.97	0.97	−0.4 ± 6.1	excellent	0.96	0.96	0.91
	lateral flexion	Fast	excellent	0.94	3.6	0.99	0.99	5.9 ± 8.3	good	0.95	0.97	0.94
		Slow	excellent	0.94	4.1	0.99	0.99	6.1 ± 8.6	excellent	0.96	0.95	0.92
	rotation	Fast	excellent	0.94	3.9	0.99	0.98	5.0 ± 8.6	good	0.91	0.95	0.92
		Slow	excellent	0.90	3.8	0.99	0.98	4.4 ± 6.9	excellent	0.93	0.94	0.86

**Table 2:** The statistical metrics of validity and reliability of the PNS in the complex task.

Variable			Validity						Reliability			
			Degree of Validity	CMC	RMSE (°)	Pearson’s r	R^2^	LOA (°)	Degree of Reliability	ICC (Intrasession)	ICC (Intersession)	CMC (Intersession)
Shoulder	flexion/ extension	Fast	excellent	0.98	7.5	0.98	0.96	25.9 ± 6.4	excellent	0.92	0.97	0.96
		Slow	excellent	0.98	8.8	0.97	0.96	17.9 ± 11.9	excellent	0.95	0.97	0.97
	adduction/ abduction	Fast	good	0.89	5.0	0.88	0.80	−12.6 ± 16.0	good	0.87	0.87	0.88
		Slow	good	0.89	6.8	0.90	0.81	−11.3 ± 13.6	good	0.86	0.86	0.84
	internal/ external rotation	Fast	good	0.88	9.0	0.87	0.79	−13.0 ± 15.9	good	0.87	0.85	0.82
		Slow	good	0.88	8.2	0.86	0.79	−11.5 ± 17.7	good	0.89	0.87	0.86
Elbow	flexion/ extension	Fast	good	0.95	12.3	0.97	0.95	27.8 ± 18.8	good	0.86	0.92	0.89
		Slow	good	0.92	12.5	0.95	0.94	27.1 ± 16.1	excellent	0.90	0.95	0.95
	pronation/ supination	Fast	excellent	0.91	9.3	0.89	0.81	8.3 ± 6.9	good	0.90	0.88	0.84
		Slow	excellent	0.92	9.5	0.90	0.81	6.6 ± 7.2	good	0.88	0.91	0.79
Thorax	flexion/ extension	Fast	excellent	0.96	10.0	0.97	0.97	−6.2 ± 21.7	excellent	0.90	0.96	0.95
		Slow	excellent	0.96	10.4	0.97	0.97	−3.7 ± 24.8	excellent	0.94	0.97	0.96
	lateral flexion	Fast	excellent	0.91	2.2	0.91	0.84	2.5 ± 5.1	good	0.89	0.93	0.87
		Slow	excellent	0.93	2.1	0.92	0.85	1.5 ± 4.2	excellent	0.90	0.93	0.92
	rotation	Fast	excellent	0.93	1.9	0.93	0.87	−0.7 ± 1.3	good	0.88	0.96	0.94
		Slow	excellent	0.95	2.1	0.95	0.91	−0.2 ± 2.3	good	0.86	0.93	0.89

**Table 3:** Paired *t*-test for validity metrics between task complexities and between movement speeds.

	CMC	Pearson’s r	R^2^	RMSE	Bias
Task complexities					
Simple task	0.92 ± 0.04	0.97 ± 0.03	0.96 ± 0.04	6.38 ± 2.29	7.79 ± 6.18
Complex task	0.93 ± 0.03	0.93 ± 0.04	0.88 ± 0.07	7.35 ± 3.74	11.05 ± 9.52
*p* value	0.838	0.029	0.015	0.459	0.363
Effect size of Cohen’s d	0.075	0.969	1.14	0.277	0.344
Movement speeds					
Fast speed	0.93 ± 0.03	0.95 ± 0.03	0.91 ± 0.05	6.82 ± 2.63	10.08 ± 6.71
Slow speed	0.93 ± 0.03	0.94 ± 0.02	0.92 ± 0.04	6.91 ± 2.53	8.77 ± 6.33
*p* value	0.712	0.474	0.150	0.691	0.046
Effect size of Cohen’s d	0.136	0.268	0.572	0.146	0.855

## Data Availability

The data presented in this study are openly available in the FigShare repository at https://doi.org/10.6084/m9.figshare.20633139.

## Appendix A

**Table A1:** Typical error of measurement, SWC0.2; 06; and 1.2 and MDC95% of intertest session.

Variable		TEM	SWC_0.2_	SWC_0.6_	SWC_1.2_	MDC_95%_
Shoulder	flexion/ extension	0.77	1.14	3.41	6.81	2.13
	adduction/ abduction	1.26	1.21	3.63	7.25	3.49
	internal/ external rotation	2.53	1.61	4.82	9.65	7.03
Elbow	flexion/ extension	1.85	3.05	9.16	18.31	5.12
	pronation/ supination	3.31	2.88	8.65	17.30	9.18
Thorax	flexion/ extension	1.63	2.86	8.58	17.17	4.52
	lateral flexion	1.25	0.97	2.92	5.84	3.47
	rotation	0.48	0.78	2.34	4.68	1.34
